# Health-related quality of life for First Nations and Caucasian women in the First Nations Bone Health Study

**DOI:** 10.1186/s13104-017-3081-z

**Published:** 2017-12-20

**Authors:** Lana G. Tennenhouse, William D. Leslie, Lisa M. Lix

**Affiliations:** 1 George and Fay Yee Centre for Healthcare Innovation, University of Manitoba, Winnipeg, MB Canada; 2 Department of Internal Medicine, Rady Faculty of Health Sciences, University of Manitoba, Winnipeg, MB Canada; 3 Department of Community Health Sciences, University of Manitoba, Winnipeg, MB Canada

**Keywords:** Indigenous, Health-related quality of life, Determinants of health

## Abstract

**Objective:**

Studies about the health of Indigenous (i.e., original inhabitants) populations often focus on chronic diseases and risk behaviors, emphasizing physical aspects of health. Our objective was to test for differences in self-reported health-related quality of life (HRQOL), which provides a multidimensional and holistic perspective on health, between First Nations (one group of Indigenous peoples) and Caucasian women. Data were from the First Nations Bone Health Study, conducted in the Canadian province of Manitoba. HRQOL was measured using the validated Medical Outcomes Study 36-Item Short Form Health Survey (SF-36). It captures respondent’s perceptions of eight health domains, as well as overall mental and physical health components.

**Results:**

Analyses were conducted for 707 participants of which 47.4% were of First Nations origin. First Nations respondents had significantly lower unadjusted scores (p < 0.05) than Caucasian respondents on all SF-36 dimensions, except bodily pain and vitality. They also had significantly lower overall mental health scores. After adjusting for multiple determinants of health (e.g., age, education, substance use), differences were no longer statistically significant, except for the social functioning and role emotional domains and overall mental health component. Complex cultural factors are likely responsible for the persistent mental health inequalities experienced by First Nations women.

**Electronic supplementary material:**

The online version of this article (10.1186/s13104-017-3081-z) contains supplementary material, which is available to authorized users.

## Introduction

Indigenous peoples of Canada are the original inhabitants of Canada; they include First Nations, Inuit, and Métis peoples and comprise 4% of Canada’s population [1]. Many studies about the health of Indigenous (also referred to as Aboriginal) populations focus on chronic diseases, such as diabetes, and health risk behaviors, such as smoking, which are often more prevalent in Indigenous peoples than in other Canadians [2–4]. Health-related quality of life (HRQOL) measures may be a better choice to describe the health of Indigenous peoples because they provide a multidimensional perspective on self-perceived physical and mental health [5, 6] and insights about functional status and well-being. This holistic approach to health coincides with Indigenous ideas that focus on physical, mental, emotional, and spiritual aspects of the individual [7–9].

Studies about the HRQOL of Indigenous populations or differences in HRQOL for Indigenous and non-Indigenous populations have primarily been conducted in the United States [10]. For example, Zack found that American Indians and Alaskan Native adults were more likely to report lower self-rated health and higher numbers of physically and mentally unhealthy days, even after adjusting for age differences [11]. Few studies about the HRQOL of Indigenous peoples have been conducted in Canada [10]. Barton et al. compared the overall HRQOL of Aboriginal and non-Aboriginal individuals from one community in British Columbia; but Aboriginal participants only included residential school survivors. The authors found that Aboriginal respondents reported poorer self-perceived health than non-Aboriginal respondents [12].

In this research, we examined HRQOL in First Nations and Caucasian women from the First Nations Bone Health Study, a population-based cohort study from the central Canadian province of Manitoba [13–16]. This cohort study was originally conducted to investigate indicators of bone health in Canadian First Nations and Caucasian women. A prior study with these data found that HRQOL measures exhibited measurement equivalence, that is, that First Nations and Caucasian respondents interpreted questions about their HRQOL in equivalent ways [17]. However, differences in HRQOL between these two groups were not systematically explored. Our objectives were to test for differences in HRQOL between First Nations and Caucasian women and to examine the effect of demographic, socioeconomic, and health characteristics on differences in HRQOL.

## Main text

### Data source

Study participants in the First Nations Bone Health Study consist of urban and rural First Nations and Caucasian female residents of the province of Manitoba. Recruitment occurred in two waves, with the first occurring from June 2002 to March 2004 and the second from September 2005 to August 2011.

Urban women (operationally defined as residing within 50 km of the provincial capital of Winnipeg) were identified from the population registry maintained by the provincial Ministry of Health. First Nations women were identified from national and provincial registry files. For each age and ethnic stratum, a random sample of participants was selected. A letter introducing the study was mailed to participants, with an invitation to contact research personnel for more information and to enlist. The mailing originated from the provincial ministry of health and the names of people receiving letters were anonymous to researchers until the potential participant contacted study personnel. All unused envelopes were destroyed and it was therefore not possible to determine the response rate.

For the rural sample, women residing in two First Nations communities were contacted during the first wave of the study. In the second wave, First Nations and Caucasian women were recruited from communities with large proportions of individuals from both groups in close geographic proximity to one another. For First Nations women, a random sample of participants was selected using membership lists from the communities; local community workers contacted the participants. For women living outside of these communities, recruitment leaflets and bulletins were distributed by the postal service.

An Indigenous research nurse coordinated recruitment and data collection for urban participants and trained community workers who were hired to assist in recruitment and data collection from the rural Indigenous communities. Individuals who did not speak English or a major Indigenous dialect were excluded. Self-identification of ethnic origin was confirmed at the initial participant contact and again at study enrollment.

### Study measures

All study participants completed an interviewer-administered survey adapted from the Canadian Multicentre Osteoporosis Study (CaMos), a prospective cohort study about the distribution and impact of osteoporosis in Canada [18]. The Medical Outcomes Study 36-Item Short Form Health Survey (SF-36) was used to measure HRQOL; it encompasses eight domains of health, including physical functioning, role physical, bodily pain, general health, vitality, social functioning, role emotional and mental health. As well, scale scores were aggregated to produce physical and mental component summaries. Higher scores correspond to better HRQOL. Norm-based scoring described by Ware et al. [19, 20] was conducted. The SF-36 normative data for Canadian females used in the scoring process was obtained from Hopman et al. [21].

Demographic, socioeconomic, and health characteristics of the study participants were used to define the following covariates: (a) age (25–39 years, 40–59 years, 60–75 years), (b) region of residence (rural south, rural north, urban), (c) annual household income (< $25,000, $25,000–$54,999, $55,000 or higher), (d) highest level of educational attainment (less than grade 9, grade 9 to 13 without certificate/diploma, high school diploma/certificate, university, trades, or professional certificate/diploma/degree), (e) employment status (full time, other), (f) body mass index (< 25.0, 25.0–29.9, 30.0 +), (g) alcohol consumption of more than seven drinks per week (yes, no), (h) daily cigarette usage for at least 6 months (yes, no), (i) participation in regular physical activity (yes, no), and (j) diagnoses (yes, no) of diabetes and bone-related conditions (i.e., osteoporosis, rheumatoid arthritis, osteoarthritis, and any other reported bone conditions). Data on these characteristics were self-reported, except for body mass index, which was calculated from measured height and weight.

### Statistical analysis

Frequencies and percentages were used to describe the characteristics of the study cohort and differences between First Nations and Caucasian women were assessed with χ^2^ tests of independence. Multiple linear regression models were used to test the association of ethnic origin with HRQOL for each SF-36 domain and the two summary components. We considered two models: (a) intercept only (i.e., unadjusted), and (b) adjusted for demographic, socioeconomic, and health characteristics. The *R* ^2^ statistic was used to describe total variation in the data explained by the fitted model. The regression models were assessed using scatterplots of the residuals and variance inflation factors. Individuals with missing observations were excluded.

The SF-36 domain and component summary scores were described with unadjusted and adjusted means, 95% confidence intervals (CIs), and p values. All analyses were conducted using R software version 3.3.0. Statistical significance was evaluated using *α* = 0.05.

### Results

Overall, 707 study participants had complete data on all study measures (47.4% First Nations). The amount of missing data was small and resulted in exclusion of only 37 individuals; almost equal numbers were First Nations and Caucasian (see Additional file 1). The statistical assumptions of the regression analyses were satisfied and covariate collinearity was not observed.

There were statistically significant differences in demographic characteristics between the two groups (Table 1). First Nations women were younger than their Caucasian counterparts (p < 0.001) and more First Nations than Caucasian women were recruited from urban areas (p < 0.001).

**Table 1:** Characteristics of First Nations Bone Health Study participants

Characteristic	First Nations (*N* = 335) *n* (%)	Caucasian (*N* = 372) *n* (%)	*p* value
Age (years)			< *0.001*
25–39	142 (42.4)	116 (31.2)	
40–59	136 (40.6)	154 (41.4)	
60–75	57 (17.0)	102 (27.4)	
Region of residence			< *0.001*
Urban	212 (63.3)	182 (48.9)	
Rural, south	61 (18.2)	87 (23.4)	
Rural, north	62 (18.5)	103 (27.7)	
Highest level of completed education			< *0.001*
< Grade 9	38 (11.3)	12 (3.2)	
Grade 9–13, without certificate/diploma	89 (26.6)	65 (17.5)	
High school certificate/diploma	42 (12.5)	65 (17.5)	
University, trades, or professional certificate/diploma/degree	166 (49.6)	230 (61.8)	
Employed full time	169 (50.5)	181 (48.7)	0.69
Annual household income			< *0.001*
< $25,000	119 (35.5)	50 (13.4)	
$25,000–$54,999	112 (33.4)	140 (37.6)	
≥ $55,000	67 (20.0)	155 (41.7)	
Don’t know	37 (11.0)	27 (7.3)	
Body mass index			*0.001*
< 25.0 (underweight or normal weight)	63 (18.8)	112 (30.1)	
25.0–29.9 (overweight)	104 (31.0)	112 (30.1)	
≥ 30.0 (obese)	168 (50.2)	148 (39.8)	
Participation in regular physical activity	96 (28.7)	169 (45.4)	< *0.001*
Substance use			
Alcohol usage (> 7 drinks per week)	13 (3.9)	12 (3.2)	0.79
Cigarette usage (> 6 months)	248 (66.7)	191 (51.3)	< *0.001*
Medical conditions			
Diabetes	67 (20.0)	22 (5.9)	< *0.001*
Bone-related conditions	85 (25.4)	110 (29.6)	0.25

Bone-related conditions include osteoporosis, rheumatoid arthritis, osteoarthritis, and other self-reported bone conditions. Italic p values are statistically significant at α = 0.05

Other characteristics of the two groups also differed (Table 1). First Nations women were more likely to be in lower income categories (p < 0.001) and had lower educational attainment (p < 0.001). More First Nations women than Caucasian women had high body mass index values (p = 0.0012), smoked cigarettes daily for at least 6 months (p < 0.001), and had been diagnosed with diabetes (p < 0.001). First Nations women were also less likely to participate in regular physical activity (p < 0.001).

**Fig. 1:**
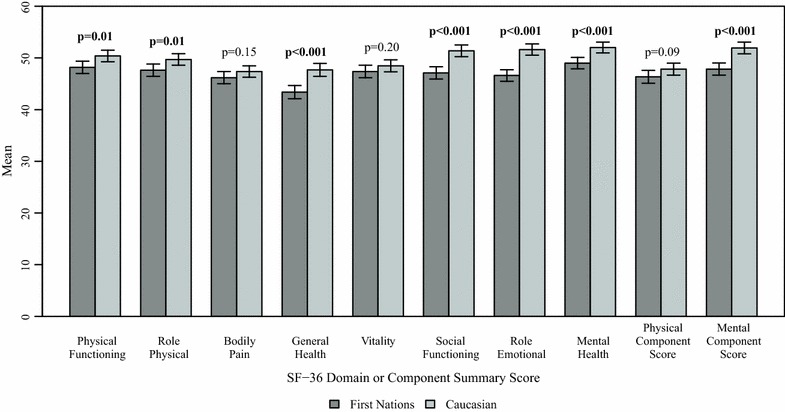
Unadjusted means (± 95% confidence intervals) for SF-36 domain and component scores, stratified by ethnicity. p values are for tests of differences in unadjusted means; bold values are statistically significant at α = 0.05

**Fig. 2:**
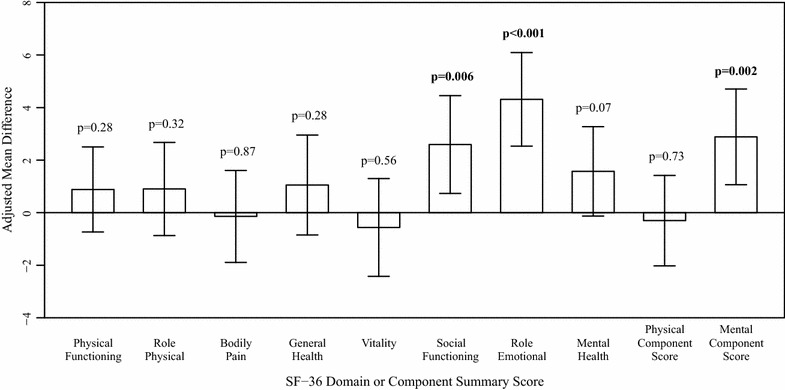
Differences in adjusted means (± 95% confidence intervals) for SF-36 domain and component scores. Differences are Caucasian—First Nations

First Nations women had significantly lower HRQOL than Caucasian women in the intercept-only regression models for all SF-36 domains and components, with the exception of the bodily pain and vitality domains and the physical health component (Fig. 1). After adjusting for covariates, the differences in scores for the physical functioning, role physical, and general health domains were no longer statistically significant (Fig. 2). Caucasian women had significantly higher adjusted scores than First Nations women on social functioning (Adjusted mean difference = 2.59, 95% CI 0.73–4.45, p = 0.006) and role emotional (Adjusted mean difference = 4.31, 95% CI 2.53–6.09, p < 0.001) domains, and on the mental health component (Adjusted mean difference = 2.88, 95% CI 1.06–4.70, p = 0.002).

The correlates of the physical and mental component summary scores are reported in Additional file 2. Poor physical health was associated with older age, urban residence, not participating in regular physical activities, obesity, bone-related conditions and diabetes. Poor mental health was associated with younger age, urban residence, not being employed full-time, and consumption of more than seven alcoholic beverages per week. The covariate-adjusted regression models explained between 7 and 30% of the variation in SF-36 domain and component scores.

### Discussion

First Nations women had mean scores below 50 for all SF-36 domains and components, which indicates that they were scoring lower than the published normative SF-36 values for Canadian women. These results are consistent with previous Canadian studies [12, 22], and suggest relatively low levels of well-being and functional status experienced by Indigenous women in Canada. While this result might be explained by greater chronic disease and health risk behaviors in Indigenous peoples, the presence of comorbidities does not always correlate with low quality of life [23, 24].

Caucasian women had higher unadjusted scores on many of the SF-36 domains related to physical health, as well as on the physical component summary score. However, these differences were no longer statistically significant after adjustment for socio-demographic and health characteristics. Higher rates of obesity, diabetes, bone-related conditions, and lower levels of physical activity in First Nations women were largely responsible for these differences. These findings reinforce the importance of diet and exercise in maintaining good health. Interventions to overcome barriers to nutritious and culturally-appropriate food [25] and physical activity [26] may therefore have a large impact on the physical well-being of First Nations women.

First Nations women experienced poorer social functioning, role emotional, and overall mental health component scores, which persisted after adjustment for multiple determinants of health. One potential explanation for these differences is that we could not adjust for risk factors that may be associated with mental health. In a study of Aboriginal communities in Ontario and British Columbia, Kant et al. [9] found that social, cultural, and land use (SCLU) factors such as access to cultural activities and lands were important indicators of well-being and that traditional Western notions of socioeconomic status were not associated as strongly with health as SCLU factors. This suggests complex cultural factors underlie mental health inequalities experienced by First Nations women, and that investigators should not assume that risk factors for poor mental health are the same for all ethnic groups. A focus on Indigenous views of health and life experiences in future studies may provide further insights about the persistent mental health inequalities between First Nations and Caucasian women.

## Limitations

These results should be considered in the context of our study’s limitations. We did not have the data required to determine the study response rate, which makes it difficult to assess the potential influence of selection bias. Our study was limited to women; the results may not generalize to males. We focused on First Nations and Caucasian individuals from one Canadian province; the results may not generalize to the populations of other provinces or to Indigenous populations in other countries. The regression models were not able to explain a substantial portion of the total variance in SF-36 scores, though this was not surprising given that there are many factors that could influence HRQOL and that were not included in this study. While previous research has demonstrated measurement equivalence of the SF-36 in First Nations and Caucasian women [17], it is important to consider that HRQOL is a subjective experience and its interpretation may be influenced by one’s ethnic origin. Internationally, investigators are collaborating with Indigenous peoples to construct and adapt HRQOL measures to optimally reflect their perspectives [10, 27]. To our knowledge, however, none of these measures have been used or validated in Canadian populations.

## Additional files

**Additional file 1.** Flow chart for study inclusion, First Nations Bone Health Study.

**Additional file 2.** Estimated regression coefficients (95% CI) and p-values for models of physical and mental component summary scores, First Nations Bone Health Study.

## Abbreviations

HRQOL: health-related quality of life
SF-36: Medical Outcomes Study 36-Item Short Form Health Survey
