# Supplementary material for: Health-related quality of life for First Nations and Caucasian women in the First Nations Bone Health Study
Source: BMC Res Notes. 2017 Dec 20;10:755. doi: 10.1186/s13104-017-3081-z (PMC5738740; doi:10.1186/s13104-017-3081-z)
Supplement: Supplementary file 1 — Additional file 1. Flow chart for study inclusion, First Nations Bone Health Study. [file 13104_2017_3081_MOESM1_ESM.docx]

37 excluded because of missing data (19 First Nations & 18 Caucasian)

707 study participants retained in the study cohort

744 participants in the First Nations Bone Health Study (354 First Nations & 390 Caucasian)

335 First Nations

372 Caucasian
