# Supplementary material for: Health-related quality of life for First Nations and Caucasian women in the First Nations Bone Health Study
Source: BMC Res Notes. 2017 Dec 20;10:755. doi: 10.1186/s13104-017-3081-z (PMC5738740; doi:10.1186/s13104-017-3081-z)
Supplement: Supplementary file 2 — Additional file 2. Estimated regression coefficients (95% CI) and p-values for models of physical and mental component summary scores, First Nations Bone Health Study. [file 13104_2017_3081_MOESM2_ESM.docx]

| Variable | Physical Component Summary | | Mental Component Summary | |
| --- | --- | --- | --- | --- |
|  | Coefficient  (95% CI) | p-value | Coefficient  (95% CI) | p-value |
| *Ethnicity* |  |  |  |  |
| Caucasian | REF |  | REF |  |
| First Nations | 0.30 (-1.41, 2.02) | 0.73 | **-2.88 (-4.71, -1.06)** | **0.002** |
| *Age* |  |  |  |  |
| 25-39 years | REF |  | REF |  |
| 40-59 years | -1.71 (-3.51, 0.09) | 0.06 | **3.34 (1.42, 5.25)** | **0.001** |
| 60-75 years | -1.96 (-4.41, 0.49) | 0.12 | **6.82 (4.22, 9.42)** | **<0.001** |
| *Region of Residence* |  |  |  |  |
| Urban | REF |  | REF |  |
| Rural, South | **2.46 (0.52, 4.39)** | **0.01** | **2.96 (0.90, 5.02)** | **0.005** |
| Rural, North | 1.85 (-0.13, 3.83) | 0.07 | 0.70 (-1.40, 2.81) | 0.66 |
| *Highest level of completed education* |  |  |  |  |
| <Grade 9 | REF |  | REF |  |
| Grade 9-13, without certificate/diploma | 2.41 (-0.98, 5.80) | 0.16 | 2.20 (-1.40, 5.80) | 0.23 |
| High school certificate/diploma | 3.40 (-0.29, 7.08) | 0.07 | 1.58 (-2.34, 5.50) | 0.43 |
| University, trades, or professional certificate/diploma/degree | 2.71 (-0.68, 6.10) | 0.12 | 1.85 (-1.76, 5.45) | 0.31 |
| *Employed full time* | 0.59 (-1.25, 2.43) | 0.53 | **3.07 (1.11, 5.02)** | **0.002** |
| *Annual household income* |  |  |  |  |
| <$25,000 | REF |  | REF |  |
| $25,000–$54,999 | 1.13 (-1.02, 3.27) | 0.30 | 0.27 (-2.01, 2.56) | 0.82 |
| ≥$55,000 | 2.13 (-0.35, 4.61) | 0.09 | 0.49 (-2.15, 3.13) | 0.72 |
| Don't know | 2.19 (-0.76, 5.13) | 0.15 | 1.19 (-1.94, 4.32) | 0.46 |
| *Body mass index* |  |  |  |  |
| <25.0 (underweight or normal weight) | REF |  | REF |  |
| 25.0-29.9 (overweight) | -1.79 (-3.82, 0.24) | 0.08 | 1.45 (-0.71, 3.62) | 0.19 |
| ≥30.0 (obese) | -4.57 (-6.50, -2.63) | **<0.001** | 0.12 (-1.94, 2.18) | 0.92 |
| *Participation in regular physical activity* | 2.23 (0.65, 3.81) | **0.01** | -0.38 (02.06, 1.30) | 0.65 |
| *Substance use* |  |  |  |  |
| Alcohol use (> 7 drinks per week) | 3.69 (-0.38, 7.75) | 0.08 | **-5.35 (-9.67, -1.03)** | **0.02** |
| Cigarette usage (> 6 months) | 0.19 (-1.41, 1.79) | 0.82 | 0.28 (-1.42, 1.97) | 0.75 |
| *Medical conditions* |  |  |  |  |
| Diabetes | **-4.16 (-6.62, -1.71)** | **0.001** | -1.72 (-4.33, 0.89) | 0.20 |
| Bone-related conditions | **-8.81 (-10.61, -7.02)** | **<0.001** | 0.15 (-1.76, 2.06) | 0.88 |
| *R*^2^ Statistic | 0.27 |  | 0.11 |  |

Note: CI = Confidence interval; Bold values indicate a coefficient that is statistically significant at *α* = 0.05
